# A new tumour suppression mechanism by p27^Kip1^: EGFR down-regulation mediated by JNK/c-Jun pathway inhibition

**DOI:** 10.1042/BJ20140103

**Published:** 2014-10-10

**Authors:** Yong Fang, Yihong Wang, Yulei Wang, Yan Meng, Junlan Zhu, Honglei Jin, Jingxia Li, Dongyun Zhang, Yonghui Yu, Xue-Ru Wu, Chuanshu Huang

**Affiliations:** *Department of Medical Oncology, Sir Run Run Shaw Hospital, Zhejiang University School of Medicine, Hangzhou, Zhejiang 310016, China; †Nelson Institute of Environmental Medicine, New York University School of Medicine, Tuxedo, NY 10987, U.S.A.; ‡Department of Pathology, Sir Run Run Shaw Hospital, Zhejiang University School of Medicine, Hangzhou, Zhejiang 310016, China; §Departments of Urology and Pathology, New York University School of Medicine, New York, NY 10016, U.S.A.; ∥Veterans Affairs New York Harbor Healthcare System, Manhattan Campus, New York, NY 10010, U.S.A.

**Keywords:** bladder cancer, c-Jun N-terminal kinase (JNK)/c-Jun pathway, epidermal growth factor receptor (EGFR), p27^Kip1^, signal transduction pathway, AP-1, activator protein 1, BME, basal medium Eagle, CDK, cyclin-dependent kinase, DMEM, Dulbecco’s modified Eagle’s medium, EGFR, epidermal growth factor receptor, GAPDH, glyceraldehyde-3-phosphate dehydrogenase, HSF-1, heat-shock factor 1, Hsp, heat-shock protein, IHC, immunohistochemistry, JNK, c-Jun N-terminal kinase, MEF, mouse embryonic fibroblast, RT, reverse transcription, SP1, specificity protein 1

## Abstract

p27^Kip1^ is a potent inhibitor of cyclin-dependent kinases that drive G_1_-to-S cell-cycle transition. Reduced p27^Kip1^ expression is prevalent in a wide range of human tumours; however, the exact mechanism(s) of p27^Kip1^-mediated tumour suppression remains obscure. In the present study, we identified a close inverse relationship between p27^Kip1^ and EGFR (epidermal growth factor receptor) expression: the parental T24 human bladder cancer cells had high p27^Kip1^ expression but low EGFR expression and, in striking contrast, the metastatic derivative of T24 (T24T) had low p27^Kip1^ expression but high EGFR expression. This relationship was also found in various human cancer tissues, and was not only just correlative but also causal; depletion of p27^Kip1^ in MEF (mouse embryonic fibroblast) cells resulted in markedly elevated EGFR expression, a result reproducible with an *Egfr* promoter-luciferase reporter in both T24 and MEF cells, suggesting transcriptional repression of EGFR by p27^Kip1^. Indeed, p27^Kip1^ was found to regulate EGFR expression via the JNK (c-Jun N-terminal kinase)/c-Jun transcription factor: p27^Kip1^ deficiency activated JNK/c-Jun, whereas inhibition of JNK/c-Jun by dominant-negative mutants dramatically repressed *Egfr* transcription. Furthermore, the proximal promoter of the *Egfr* gene was crucial for its transcription, where the recruiting activity of c-Jun was much greater in p27^Kip1−/−^ cells than in p27^Kip1+/+^ cells. Introduction of GFP–p27^Kip1^ into T24T cells suppressed JNK/c-Jun activation, EGFR expression and anchorage-independent growth. The results of the present study demonstrate that p27^Kip1^ suppresses JNK/c-Jun activation and EGFR expression in MEFs and human bladder cancer cells, and the results obtained are consistent with those from human cancer specimens. The present study provides new insights into p27^Kip1^ suppression of cancer cell growth, migration and metastasis.

## Abstract 2

An inverse relationship between p27^Kip1^ and EGFR expression in parental T24 human bladder cancer cells and various human cancer tissues was found. Depletion of p27^Kip1^ in cells markedly elevated EGFR expression through transcriptional repression of *Egfr* by p27^Kip1^ via the JNK/c-Jun cascade.

## INTRODUCTION

p27^Kip1^, encoded by the *CDKN1B* gene, is a strong inhibitor of the CDKs (cyclin-dependent kinases) that propel the progression of the cell cycle from G_1_- to S-phase [1]. When overexpressed in cultured cells, p27^Kip1^ causes G_1_ arrest, thereby inhibiting cell growth and, conversely, depletion of p27^Kip1^ accelerates G_1_ exit [2], promoting cell proliferation [3]. In accordance with these *in vitro* attributes, loss of p27^Kip1^, along with additional genetic alterations or carcinogen exposure, predisposes mice to a wide range of tumours of both epithelial [4] and mesenchymal [5] origins. Perhaps not surprisingly, reduced expression of p27^Kip1^, as a result of transcriptional down-regulation, post-translational phosphorylation, elevated ubiquitination or nuclear-to-cytoplasmic translocation, is found in many human malignancies [6]. A decreased p27^Kip1^ protein level, in particular the nuclear fraction, also correlates well with more advanced disease stages and poorer clinical outcomes than a normal level of p27^Kip1^ in various cancers [7]. Both mouse and human data therefore strongly support the notion that p27^Kip1^ acts as a tumour suppressor [8]. Nonetheless, there have been suggestions that p27^Kip1^ is an ‘unconventional’ tumour suppressor as mutations affecting the *CDKN1B* gene are rare in human cancers [9]. However, recent identification of p27^Kip1^ mutations in breast cancer [10] and multiple endocrine neoplastic syndromes [11] raises an interesting new possibility that mutational inactivation of p27^Kip1^ in other tumour types cannot be completely ruled out [12].

Although p27^Kip1^ is a tumour suppressor and its down-regulation in tumour cells takes place on multiple levels, much less is known about precisely how p27^Kip1^ deficiency leads to disturbances in downstream effectors that are pivotal for tumorigenesis [13]. Aside from its CDK-dependent functions in tumour suppression, p27^Kip1^ has been suggested to exert CDK-independent activities [14]. A notable example is the ability of this protein to modulate the actin cytoskeleton via the regulation of RhoA activation [15]. In so doing, p27^Kip1^ can affect, albeit indirectly, cancer cell motility and migration, and in turn their propensity for invasion and metastasis [15,16]. p27^Kip1^ has also been shown to be involved in apoptosis and autophagy, although whether it is stimulatory or inhibitory might be dependent on context and experimental conditions [17]. In spite of the long-held assumption of its role, robust evidence of CDK-independent activities of p27^Kip1^ in the context of tumour initiation and promotion remains scarce.

Our group has a longstanding interest in delineating the molecular signals and pathways that distinguish advanced tumours from an early stage [18,19]. It has been proven that p27^Kip1^ could suppress arsenite-induced Hsp (heat-shock protein) 27/Hsp70 expression through inhibiting JNK (c-Jun N-terminal kinase) 2/c-Jun- and HSF-1 (heat-shock factor 1)-dependent pathways [18]. By profiling gene and protein expression of the human T24 bladder cancer cell compared with its metastatic derivative T24T, we found a striking inverse relationship between p27^Kip1^ and EGFR (epidermal growth factor receptor) expression. The relationship between p27^Kip1^ and EGFR expression remains unclear. To the best of our knowledge, this is the first demonstration that p27^Kip1^ regulates EGFR expression and that it does so by influencing JNK/c-Jun transactivation. The results of the present study expand the existing repertoire of CDK-independent activities of p27^Kip1^, and suggest that the increased cell-cycle transition due to p27^Kip1^ deficiency can synergize with increased expression of growth-promoting signals (e.g. EGFR) to accelerate tumour progression.

## EXPERIMENTAL

### Plasmids, antibodies and other reagents

The dominant-negative mutant of c-Jun (TAM67) [19] and the dominant-negative mutant of JNK (DN-JNK) [20] have been described in our previous studies [19,20]. The PRL-TK-luciferase expression vector and pSUPER vector were purchased from Promega and have been used in our previous studies [21]. Adenovirus-driven GFP (Ad-GFP) and Adenovirus-driven-GFP–p27^Kip1^ (Ad-GFP–p27) plasmids were constructed and have been used in our previous studies [18]. Constructs expressing *Egfr* promoter-driven luciferase reporter and its various deletions as indicated in Figure 2(D) were a gift from Dr D.T. Smoot (Department of Medicine and Cancer Center, Howard University, Washington, DC, U.S.A.). Antibodies against c-Jun, EGFR, GAPDH (glyceraldehyde-3-phosphate dehydrogenase), histone H3, JNK1/2, p-c-Jun Ser^63^, p-c-Jun Ser^73^ and p-JNK were purchased from Cell Signaling Technology. Antibodies against c-Fos, Fra-1, SP1 (specificity protein 1) and c-Myc were from Santa Cruz Biotechnology. The antibody against p27^Kip1^ was obtained from Abcom Biochemicals.

### Cell culture and transfection

Immortalized p27^Kip1+/+^ and p27^Kip1−/−^ MEF (mouse embryonic fibroblast) cell lines have been described in our previous studies [19,22,23]. These cells and their stable transfectants were maintained at 37°C in a 5% CO_2_ incubator with DMEM (Dulbecco's modified Eagle's medium) supplemented with 10% FBS, 2 μM L-glutamine and 25 μg/ml gentamycin. The monolayer growth of human bladder cancer T24 cells and its derived metastatic T24T cells were a gift from Dr Dan Theodorescu (University of Colorado, Denver, CO, U.S.A.) [24]. Both T24 and T24T cell lines were maintained in DMEM-F12 (1:1) (Invitrogen), supplemented with 5% heat-inactivated FBS, 2 μM L-glutamine and 25 μg/ml gentamycin. Stable cell transfectants were established by co-transfection of the indicated expression construct together with the pSUPER vector and/or the PRL-TK-luciferase expression vector using PolyJet™ DNA In Vitro Transfection Reagent (SignaGen Laboratories) by antibiotic selection, and surviving cells in each well were pooled as the stable mass transfectant as described in our previous studies [19,21,25,26].

### *Egfr* promoter-driven luciferase reporter activity assay

The *Egfr* promoter-driven luciferase reporter stable transfectant was seeded into each well of 96-well plates. Cells were collected as indicated, and then extracted with lysis buffer [25 mmol/l Tris-phosphate (pH 7.8), 2 mmol/L-EDTA, 1% Triton X-100 and 10% glycerol] for the luciferase activity assay using the luciferase assay system (Promega) together with a microplate luminometer (Microplate Luminometer LB 96V, Berthold) as described in our previous studies [27].

### IHC (immunohistochemistry) of human cancer specimens

A total of 120 patients with lung adenocarcinoma, colon cancer and bladder cancer (40 patients each) from Sir Run Run Shaw Hospital, Zhejiang University School of Medicine, were included in the present study. All participants gave written informed consent and the study was approved by an Ethics Committee Institutional Review Board of the hospital. The mean age of the patients was 54.5 years (range from 32.5 to 72.3 years); 74 cases were male. Paraffin-embedded surgery specimens were sectioned at 5 μm thickness, and mounted on to Fisher brand Super frost plus slides (Fisher Scientific). After antigen retrieval, sections were incubated overnight at 4°C with monoclonal mouse antibodies against EGFR (Abcam) or p27^Kip1^ (Zymed). IHC was carried out by using the DAB (diaminobenzidine) Map Kit (Pierce), based on the avidin–biotin complex immunoperoxidase technique. Positively stained cells were evaluated using image analysis (Image-Pro Plus, version 4.5.1, Media Cybernetics) to reduce observer variation. The expression of EGFR and p27^Kip1^ was scored based on the intensity of immunostaining as follows: count 300 cells and score ‘0’ for negative staining of the considered cells, whereas scores 1–3 present <25%, 25–50% and >50% positive staining of the considered cells respectively.

### Anchorage-independent growth assay

Anchorage-independent growth in soft agar (soft agar assay) was carried out as described in our previous studies [23]. Briefly, 1×10^4^ cells mixed with or without the JNK inhibitor SP600125 (25 μmol/l) in 10% FBS BME (basal medium Eagle) containing 0.33% soft agar, was seeded over the bottom layer of 0.5% agar in 10% FBS BME in each well of six-well plates. The plates were incubated in a 5% CO_2_ incubator at 37°C for 21 days. Colonies were visualized under a microscope and photographed. Colonies consisting of over 32 cells were counted and presented [25,28].

### Preparation of nuclear extracts

Nuclear extracts were prepared as previously described [21]. Briefly, p27^Kip1+/+^ and p27^Kip1−/−^ MEFs were plated into 10-cm culture dishes and cultured until 80% confluence. The nuclear and cytosolic proteins were extracted according to the protocol of the Nuclear/Cytosol Fractionation kit (BioVison Technologies). Protein was quantified using a protein quantification assay kit (Bio-Rad Laboratories) and then subjected to Western blot analysis.

### Western blot analysis

T24 cells, T24T cells, MEFs and their transfectants were seeded in six-well plates and cultured in normal medium containing 10% FBS until 70–80% confluence. Cells were extracted with cell lysis buffer [10 mM Tris/HCl (pH 7.4), 1% SDS and 1 mM sodium orthovanadate]. Cell extracts were subjected to Western blot analysis as described previously [18,21].

### RT (reverse transcription)–PCR

Total RNA was extracted with TRIzol® reagent (Invitrogen) and cDNAs were synthesized with the Thermo-Script RT-PCR system (Invitrogen). The amount of mRNA present in the cells was determined by semi-quantitative RT–PCR. Primers used were: 5′-GAGAGGAGAACTGCCAGA-3′ and 5′-GTAGCATTTATGGAGAGTG-3′ for the human and mouse *Egfr* gene (450 bp), 5′-ATCAAGAAGGTGGTGAAGCAGGCA-3′ and 5′-TCTCTTGCTCAGTGTCCTTGCTGGG-3′ for the mouse *Gapdh* gene (281 bp), and 5′-AGAAGGCTGGGGCTCATTTG-3′ and 5′-AGGGGCCATCCACAGTCTTC-3′ for the human *GAPDH* gene (258 bp). PCR products were separated on 2% agarose gels and stained with ethidium bromide, and the results were imaged with the Alpha Innotech SP image system as described previously [29].

### ChIP assay

The EZ-ChIP kit (Millipore Technologies) was used to carry out the ChIP assay according to the manufacturer's instructions and as described previously [30]. Briefly, p27^Kip1+/+^ and p27^Kip1−/−^ MEFs were treated with 1% formaldehyde for 10 min at room temperature. Cells were then pelleted, resuspended in lysis buffer and sonicated to generate 200- to 500-bp chromatin DNA fragments. After centrifugation (13000 ***g*** for 10 min at 4°C), a 10-fold dilution of the supernatants were incubated with an anti-c-Jun antibody or the control rabbit IgG at 4°C overnight. The immune complex was captured with Protein G–agarose-saturated beads with salmon sperm DNA and then eluted with elution buffer. The reverse cross-linking of protein–DNA complexes to free DNA was conducted by incubating at 65°C overnight. The DNA was extracted and subjected to PCR analysis. To specifically amplify the region containing the AP-1 (activator protein 1)-binding sites on the mouse *Egfr* promoter, PCR was performed with the following pair of primers: 5′-CGTGAACAGCGTCCCCACCT-3′ (from −700 to −680 bp) and 5′-GCTGTGTGCAGCGGGTCAGT-3′ (from −474 to −455 bp). PCR products were separated on 2% agarose gels and stained with ethidium bromide. The images were scanned under UV light.

### Statistical methods

Relationships between the expression of p27^Kip1^ and EGFR were analysed using Fisher's exact and Pearson's (χ^2^) tests. The Spearman test was performed to evaluate the relationship between EGFR and p27^Kip1^ expression in the IHC test. Student's *t* test was used to determine the significance of differences of *Egfr* promoter activities between groups. The differences were considered to be significant at *P*<0.05.

## RESULTS

### EGFR expression was inversely related to p27^Kip1^ expression in the T24 cell line compared with its metastatic derivative T24T cell line

**Figure 1:**
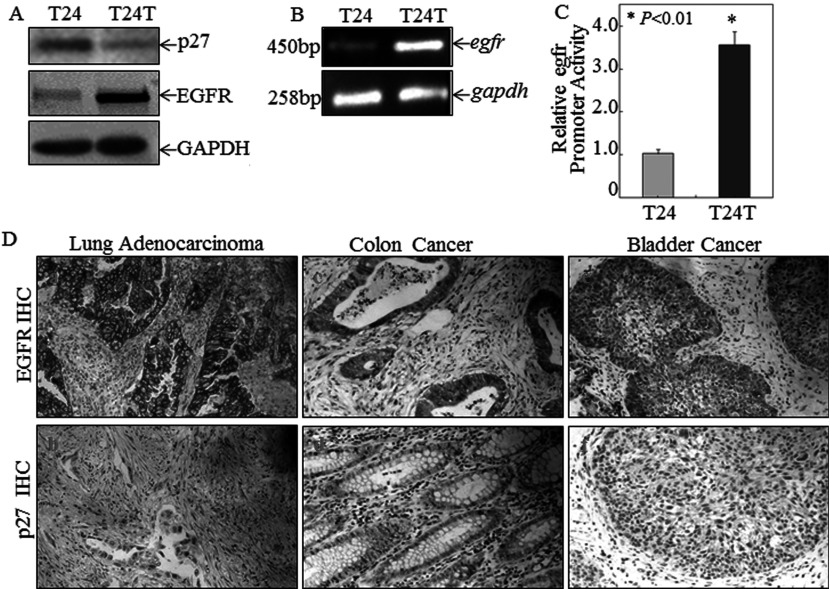
Inverse correlation of p27^Kip1^ and EGFR expression in human bladder cancer cell lines and human cancer tissues (**A**) Western blotting analysis of p27^Kip1^ and EGFR protein levels in T24 cells and their derived metastatic T24T human bladder cancer cells. GAPDH was used as loading control. (**B**) *Egfr* mRNA expression levels in T24 and T24T cells were determined by RT–PCR. *Gadph* was used as loading control. (**C**) *Egfr* promoter luciferase reporter was stably transfected into T24 and T24T bladder cancer in combination with pRL-TK used as an internal control. The results were presented as *Egfr* promoter activity in T24T cells relative to that in T24 cells. Values are means ±S.D. for triplicate assay wells. (**D**) Inverse correlation of p27^Kip1^ and EGFR expression in clinical tumour specimens detected with IHC, including in lung adenocarcinoma (*n*=40), colon cancer (*n*=40) and bladder cancer (*n*=40) (original magnification ×200).

Although EGFR is overexpressed and p27^Kip1^ is down-regulated in many human cancer types [31], the molecular interplay between the growth promoter and the tumour suppressor had not been explored [7,32]. In an effort to identify new signalling pathways that were essential for tumour progression, we first analysed divergent signalling molecules between a pair of established cell lines, e.g. T24, originally from a high-grade human bladder cancer, and T24T, a T24 derivative that has acquired the ability to metastasize [24]. Of the differentially expressed proteins, we found EGFR and p27^Kip1^ to be among the most prominent. p27^Kip1^ expression was dramatically down-regulated in T24T cells compared with its parental T24 cells, whereas EGFR was exactly the opposite, i.e. low expression in T24 cells and high in T24T cells (Figure 1A). Overexpression of EGFR in T24T cells was found to occur at the mRNA level as analysed RT–PCR (Figure 1B), a result extended by the *Egfr* promoter-driven luciferase reporter assay (Figure 1C), indicating that the differential expression of EGFR between T24 and T24T cells was regulated at the transcriptional level.

### Inverse correlation of p27^Kip1^ and EGFR expression in human cancer specimens

To further extend our findings to specimens obtained from human cancer patients, we evaluated the expression of both p27^Kip1^ and EGFR in human cancer specimens using IHC staining. As shown in Figure 1(D) and Table 1, p27^Kip1^ expression showed a significant inverse correlation with EGFR expression in lung adenocarcinoma specimens (Spearman correlation: *r_s_*=−0.360; *P*=0.019), colorectal cancer specimens (Spearman correlation: *r_s_*=−0.359; *P*=0.018) and bladder cancer specimens (Spearman correlation: *r_s_*=−0.337; *P*=0.039), strongly suggesting that p27^Kip1^ expression might be involved in the regulation of EGFR expression.

**Table 1:** The correlation between EGFR and p27 expression in human cancer specimens

		EGFR protein expression					
p27^Kip1^ protein expression	Cases	–	+	++	+++	*r_s_* value	*P* value
Lung adenocarcinoma	40					−0.360	0.019
–	23	5	6	6	6		
+	7	4	1	1	1		
++	6	2	2	1	1		
+++	4	3	1	0	0		
Colorectal adenocarcinoma	40					−0.359	0.018
–	20	7	3	6	4		
+	8	5	2	1	0		
++	7	4	2	1	0		
+++	5	2	2	1	0		
Bladder cancer	40					−0.337	0.039
–	26	6	5	6	9		
+	6	3	1	1	1		
++	5	2	2	1	0		
+++	3	1	1	1	0		

### EGFR expression was up-regulated in p27^Kip1^-knockout cells compared with their wild-type counterparts

**Figure 2:**
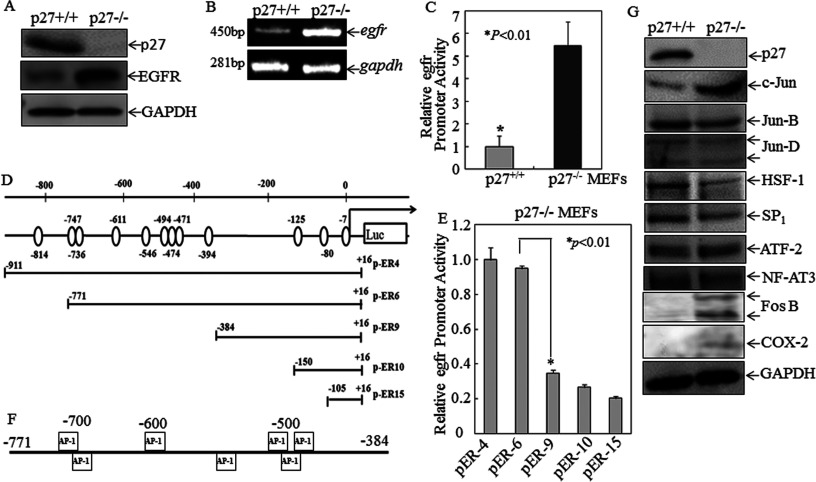
EGFR expression levels of protein, mRNA and transcription was significantly up-regulated in p27^Kip1^-knockout cells compared with wild-type counterparts, and mapping of a crucial *Egfr* promoter region for p27^Kip1^ suppression of *Egfr* transcription (**A**) Western blotting analysis of p27^Kip1^ and EGFR protein levels in p27^Kip1+/+^ compared with p27^Kip1−/−^ MEFs. GAPDH was used as a protein loading control. (**B**) *Egfr* mRNA expression levels in p27^Kip1+/+^ compared with p27^Kip1−/−^ MEFs was determined by RT–PCR. *Gadph* was used as a loading control. (**C**) *Egfr* promoter luciferase reporter plasmids were stably transfected into p27^Kip1+/+^and p27^Kip1−/−^ MEFs in combination with pRL-TK used as an internal control. Results are presented as *Egfr* promoter activity of p27^Kip1+/+^ cells relative to p27^Kip1−/−^ cells respectively. Values are means±S.D. for triplicate assay wells. (**D**) A schematic diagram of the various deletions of the *Egfr* promoter luciferase reporter with the open circles corresponding to AP-1-binding sites. The 5′ end maps to the following positions: pER4-luc (−911), pER6-luc (−771), pER9-luc (−384), pER10-luc (−150) and pER15-luc (−105). (**E**) A series of *Egfr* promoter-driven luciferase reporter stable transfectants were seeded into each well of 96-well plates. Cells were extracted with lysis buffer for a luciferase activity assay using the luciferase assay system (Promega). Results are presented as luciferase activity relative to the p27^Kip1−/−^ MEFs transfected with pER4-luc only. Values are means±S.D. for triplicate assay wells. (**F**) Bioinformatics analysis indicating transcription factor AP-1-binding sites in the *Egfr* promoter area within −771 to −384. (**G**) Others transcription factors and c-Jun target protein including COX-2 and Fos B in p27^Kip1+/+^ and p27^Kip1−/−^ MEFs were evaluated. GAPDH was used as a protein loading control.

The fact that p27^Kip1^ down-regulation in T24T cells led to EGFR overexpression strongly suggests that p27^Kip1^ plays an important role in negatively regulating EGFR expression. To establish this relationship more directly, we evaluated the effects of genetically disrupting p27^Kip1^ on EGFR expression in MEFs. As shown in Figures 2(A) and 2(B), knockout of p27^Kip1^ (p27^Kip1−/−^) resulted in increased EGFR expression at both the mRNA and protein level in comparison with p27^Kip1+/+^ cells. A similar effect occurred at the transcriptional level as evidenced by the *Egfr* promoter-driven luciferase reporter assay comparing p27^Kip1+/+^ with p27^Kip1−/−^ MEFs (Figure 2C). It is therefore clear that p27^Kip1^ has a strong inhibitory effect on EGFR transcription.

### The c-Jun-binding region in the *Egfr* promoter was crucial for p27^Kip1^ suppression of *Egfr* transcription

The fact that p27^Kip1^ inhibits *Egfr* gene transcription prompted us to perform bioinformatic profiling of the putative transcription-factor-binding motifs within the *Egfr* gene promoter using the TFSEARCH software (version 1.3). We found that the mouse *Egfr* proximal promoter contained putative DNA-binding sites for c-Jun, c-Fos, HSF-1, c-Myc, Fra-1 and SP1. To test the functional importance of these sites in p27^Kip1^-regulated *Egfr* gene transcription, we engineered truncated *Egfr* promoter regions to drive a luciferase reporter and then transfected these constructs (Figure 2D) into p27^Kip1−/−^ cells. As shown in Figure 2(E), transcriptional activity with pER9, pER10 and pER15 was significantly impaired as compared with that with pER4 and pER6, whereas there was no significant difference among transfectants pER9, pER10 and pER15. These data suggest that the −771 to −384 *Egfr* promoter region contains transcription-factor-binding site(s) important for *Egfr* transcription. As shown in Figure 2(F), the *Egfr* promoter region between −771 and −384 contained exclusively the AP-1-binding sites, suggesting that AP-1 was an important transcription factor downstream of p27^Kip1^ for p27^Kip1^-mediated transcriptional inhibition of EGFR expression. Consistently, other transcription factors and c-Jun target proteins, including COX-2 and FosB, in p27^Kip1+/+^ and p27^Kip1−/−^ MEFs were also significantly enhanced in p27^Kip1−/−^ MEFs (Figure 2G). These data show that p27^Kip1^ inhibited EGFR expression by suppressing the activation of the JNK/c-Jun pathway.

### p27^Kip1^ inhibited EGFR expression by suppressing activation of JNK/c-Jun

**Figure 3:**
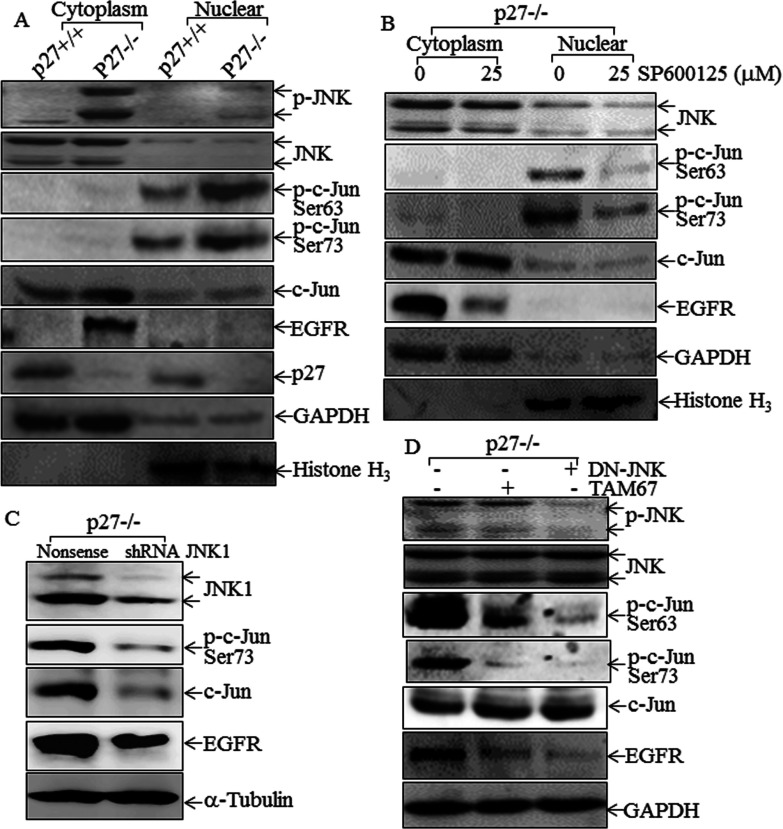
p27^Kip1^ inhibited EGFR expression by targeting JNK/c-Jun activation (**A**) Cytosolic and nuclear proteins were isolated from p27^Kip1+/+^ and p27^Kip1−/−^ MEFs with the Nuclear/Cytosol Fractionation kit. Western blotting assays were carried out to determine protein expression and phosphorylation as indicated. GAPDH and histone H3 were used as cytoplasmic and nuclear protein markers. (**B**) After pretreatment of cells with either control medium or 25 μM SP600125, cytosolic and nuclear proteins were isolated from p27^Kip1−/−^ cells using the Nuclear/Cytosol Fractionation kit. The isolated proteins were subjected to Western blotting to determine protein expression and phosphorylation as indicated. GAPDH and histone H3 were used as cytoplasmic and nuclear protein markers. (**C**) Total cell lysates from p27^Kip1−/−^ MEFs cells stably transfected with shRNA-JNK were subjected to Western blotting as indicated. α-Tubulin was used as a protein loading control. (**D**) Total cell lysates from p27^Kip1−/−^ MEFs stably transfected with either DN-JNK or TAM67 were subjected to Western blotting as indicated. GAPDH was used as a protein loading control.

**Figure 4:**
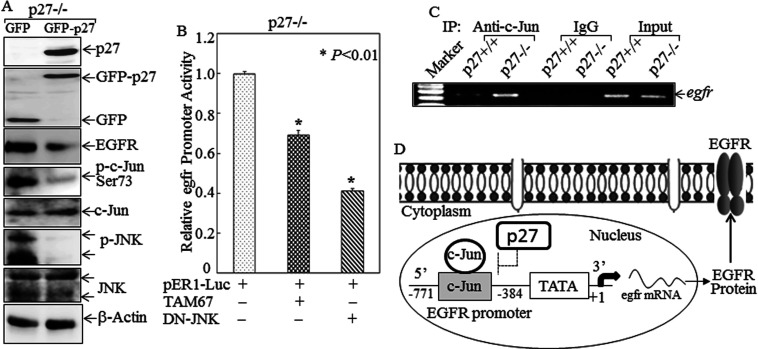
JNK/c-Jun played an essential role in *Egfr* promoter-dependent transcription activity (**A**) Either GFP or GFP–p27 plasmid was transfected into p27^Kip1−/−^ MEFs. Cells were then extracted for determination of EGFR, JNK, p-JNK, p-c-Jun and c-Jun protein levels. (**B**) The pER-1 reporter plasmid was co-transfected with either DN-JNK or TAM67 into p27^Kip1−/−^ MEFs. Cells were then extracted for determination of *Egfr* promoter-driven luciferase activity. (**C**) Soluble chromatin prepared from p27^Kip1+/+^ and p27^Kip1−/−^ MEFs was subjected to a ChIP assay using an anti-c-Jun antibody. Immunoprecipitated chromatin DNA was amplified with the PCR primers that specifically annealed to the region flanking c-Jun-responsive elements within the *Egfr* promoter. (**D**) A proposed model for p27^Kip1^ suppression of EGFR expression.

Since the *Egfr* proximal promoter contains an AP-1 site whose presence is indispensable for *Egfr* up-regulation in p27^Kip1−/−^ cells, and since c-Jun is a major AP-1 component, we determined whether c-Jun and its upstream kinase JNK were regulated by p27^Kip1^. As shown in Figure 3(A), although there was no noticeable difference in JNK protein expression and its location between p27^Kip1+/+^ and p27^Kip1−/−^ cells, JNK phosphorylation was markedly up-regulated in p27^Kip1−/−^ cells, and all phosphorylated JNK was present in the cytosolic fraction. Both c-Jun phosphorylation and protein expression were elevated in p27^Kip1−/−^ cells. Phosphorylated c-Jun was only present in the nuclear fraction, whereas non-phosphorylated c-Jun was mainly associated with the cytosolic fraction (Figure 3A). The phosphorylation of JNK and c-Jun therefore paralleled EGFR expression in p27^Kip1+/+^ compared with p27^Kip1−/−^ cells, suggesting that the JNK/c-Jun pathway might participate in p27^Kip1^ regulation of EGFR expression. In contrast with JNK and c-Jun, we observed no difference between p27^Kip1+/+^ and p27^Kip1−/−^ cells in the level, intracellular location or phosphorylation status of other transcription factors, including ATF-2 (activating transcription factor-2), HSF-1, Jun-B, Jun-D, NFAT3 (nuclear factor of activated T-cells 3) and SP1 (Figure 2G). To determine the role of JNK activation in EGFR expression in p27^Kip1−/−^ cells, we then tested a chemical inhibitor (SP600125), a dominant-negative mutant for JNK (DN-JNK), shRNA JNK and TAM67. Inhibition of JNK activation by pretreatment of p27^Kip1−/−^ cells with the JNK inhibitor SP600125 dramatically blocked both c-Jun phosphorylation and EGFR expression (Figure 3B). We further confirmed the implication of the JNK pathway in regulation of EGFR expression by using shRNA-JNK in p27^Kip1−/−^ MEFs. The results showed that the EGFR protein level decreased markedly after being transfected with shRNA-JNK, with attenuated c-Jun and p-c-Jun levels (Figure 3C). Similarly, enforced expression of DN-JNK also impaired c-Jun phosphorylation and EGFR expression in p27^Kip1−/−^ cells (Figure 3D). Furthermore, transfection with the dominant-negative mutant of c-Jun, TAM67, attenuated EGFR expression without affecting JNK phosphorylation (Figure 3D). In addition, we re-expressed p27^Kip1^ in the p27^Kip1−/−^ MEFs to confirm that the increased JNK phosphorylation and the up-regulation of EGFR are direct consequences of the absence of p27^Kip1^. As shown in Figure 4(A), either GFP–p27 or GFP plasmid was transfected into p27^Kip1−/−^ MEFs, and the results indicated that EGFR protein levels decreased greatly after being transfected with the GFP–p27 plasmid, accompanied by down-regulation of phosphorylation of p-JNK and p-c-Jun (Figure 4A). Our results clearly demonstrated that p27^Kip1^ inhibited EGFR expression by suppressing JNK/c-Jun activation.

Consistent with the effects on EGFR protein expression, enforced expression of either DN-JNK or TAM67 impaired *Egfr* promoter-driven luciferase reporter activity in p27^Kip1−/−^ cells (Figure 4B). Finally, a ChIP assay showed that recruitment of c-Jun to the *Egfr* proximal promoter was much greater in p27^Kip1−/−^cells than in p27^Kip1+/+^ cells (Figure 4C). Since c-Jun is an obligate member of the AP-1 protein dimer that binds to this *Egfr* promoter region, our studies provide compelling evidence to indicate that activation of the JNK/c-Jun pathway increases c-Jun binding to the *Egfr* promoter and up-regulates EGFR transcription and expression in p27^Kip1−/−^ cells. Our results suggest that under normal circumstances p27^Kip1^ inhibits EGFR transcription and expression via inhibition of the JNK/c-Jun axis as shown in schematic form in Figure 4(D).

### Down-regulation of p27^Kip1^ induced JNK/c-Jun phosphorylation, EGFR up-regulation and anchorage-independent growth elevation

**Figure 5:**
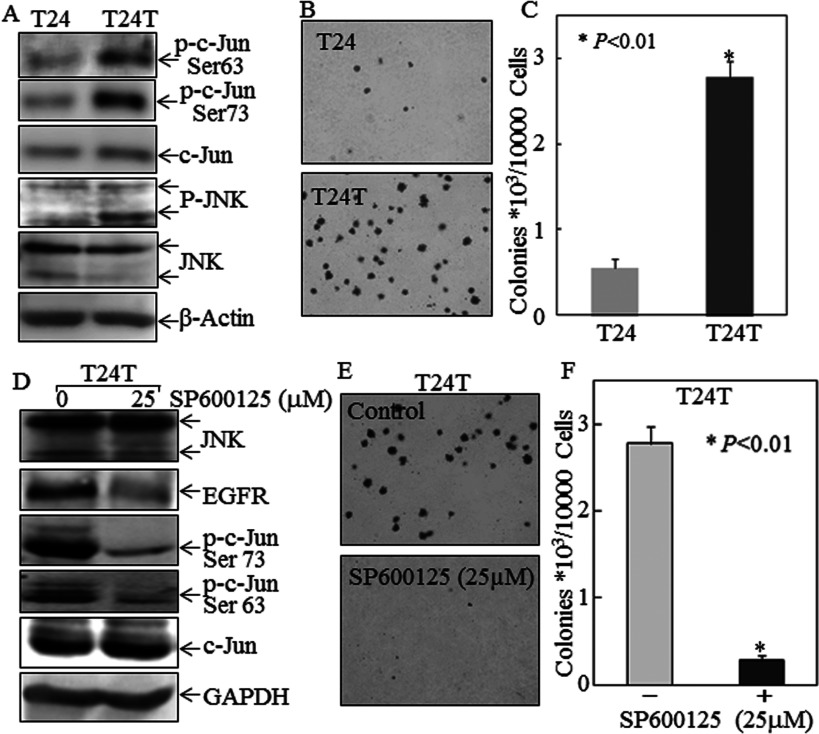
Low p27^Kip1^ expression in T24T cells mediated JNK/c-Jun phosphorylation, EGFR up-regulation and increased anchorage-independent growth (**A**) Total cell lysates from T24 and T24T bladder cancer cells were subjected to Western blotting as indicated. β-Actin was used as a protein loading control. (**B** and **C**) Anchorage-independent growth of T24 and T24T cells was determined by soft agar assay. Colonies were photographed and counted, and values are means±S.D. for triplicate assays. (**D**) T24T cells were pretreated with or without SP600125, and then extracted for determination of protein expression by Western blotting. (**E** and **F**) T24T cells were pretreated with 25 μM SP600125 for 4 h and then subjected to anchorage-independent growth. Colonies were photographed and counted. The asterisk (*) indicates a significant decrease in anchorage-independent growth of T24T pretreated with SP600125 compared with T24T cells pretreated with 0.1% DMSO control (*P*<0.01).

**Figure 6:**
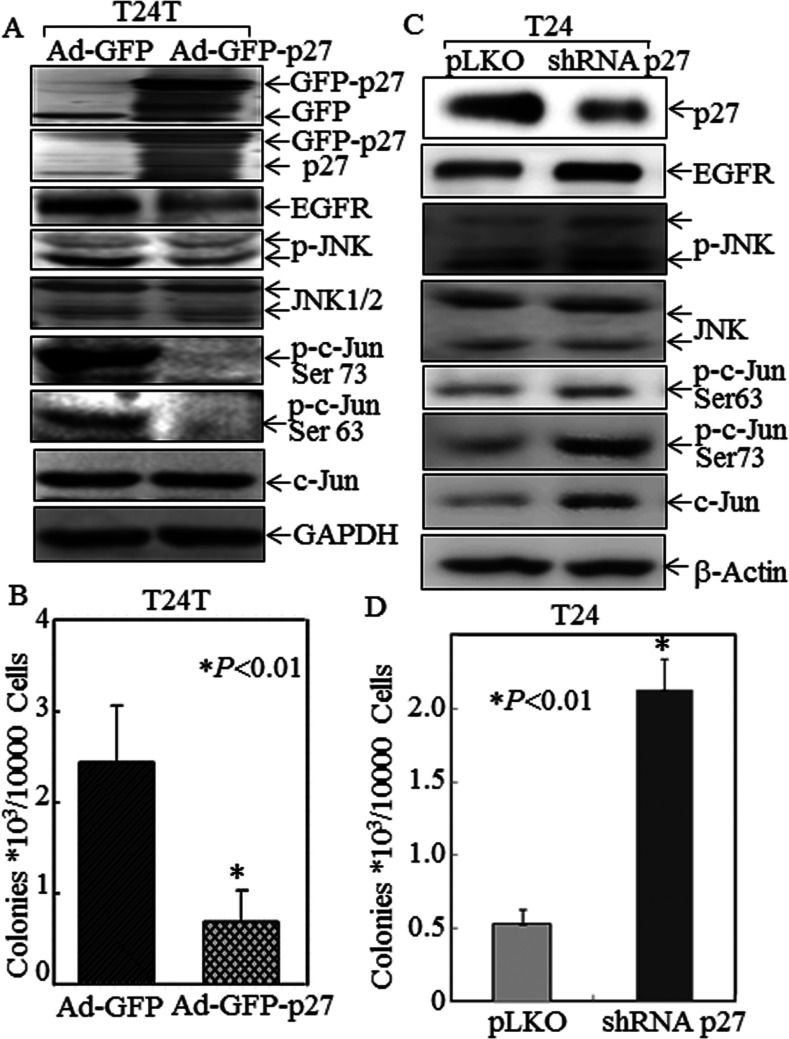
Inhibitory effect of p27^Kip1^ on JNK/c-Jun phosphorylation, EGFR expression and anchorage-independent growth in T24 and T24T cells (**A**) T24T cells were infected with Ad-GFP or Ad-GFP–p27, and then extracted for determination of protein expression by Western blotting. (**B**) T24T cells were infected with Ad-GFP or Ad-GFP–p27, and then subjected to determination of anchorage-independent growth in soft agar assay. The asterisk (*) indicates a significant decrease in anchorage-independent growth of T24T cells infected with Ad-GFP–p27 compared with those infected with Ad-GFP (*P*<0.01). (**C**) T24 cells were stably transfected with shRNA p27 and the cell extracts were subjected to Western blotting for determination of p27 knockdown in regulating JNK, p-c-Jun and EGFR expression, as compared with those transfected with control vector pLKO. (**D**) T24 cells were stably transfected with shRNA p27 or pLKO and then subjected to anchorage-independent growth. The asterisk (*) indicates a significant increase in anchorage-independent growth of T24 cells transfected with shRNA p27 compared with T24 cells transfected with pLKO (*P*<0.01).

Compared with T24 cells, there was a reduction in p27^Kip1^ expression and up-regulation of EGFR expression in T24T cells (Figures 1A and 1B). To understand the biological consequences of p27^Kip1^-regulated EGFR expression, we also compared c-Jun and JNK activation as well as the anchorage-independent growth between T24 and T24T cells. As shown in Figure 5(A), the phosphorylation of JNK and c-Jun are both up-regulated in T24T cells. Consistently, an increase in anchorage-independent growth in T24T cells was also observed as compared with T24 cells (Figures 5B and 5C). Inhibition of JNK activation by pretreatment of T24T cells with the JNK inhibitor SP600125 blocked both c-Jun phosphorylation at Ser^63^/Ser^73^ and anchorage-independent growth of T24T cells (Figures 5D–5F). To provide direct evidence that p27^Kip1^-regulated EGFR expression via the JNK/c-Jun axis plays a role in cancer cell growth, we infected T24T cells with an adenovirus-driven-GFP–p27^Kip1^ and adenovirus-driven GFP as a negative control. Enforced expression of GFP–p27^Kip1^ in T24T cells dramatically inhibited the activation of JNK and c-Jun, expression of EGFR and anchorage-independent growth (Figures 6A and 6B). Moreover, knockdown of p27^Kip1^ in T24 cells led to an increased EGFR expression and anchorage-independent growth with induction of JNK and c-Jun activation (Figures 6C and 6D). Our results demonstrate that p27^Kip1^ down-regulation plays a key role in up-regulating the JNK/c-Jun pathway, leading to increased EGFR expression and anchorage-independent growth and endowing these cancer cells with a new metastatic potential.

## DISCUSSION

A principal finding we made in the present study is that p27^Kip1^ deficiency leads to a marked transcriptional up-regulation of EGFR expression. This finding is based on several independent lines of experimental evidence. First, an inverse relationship exists between p27^Kip1^ and EGFR levels, i.e. the T24 cell line that expresses a high level of p27^Kip1^ has a low level of EGFR, and the isogenic T24T cell that expresses a low level of p27^Kip1^ has a high level of EGFR. Secondly, an inverse correlation of p27 and EGFR expression was observed in clinical human cancer tissues. Thirdly, depletion of p27^Kip1^ in p27^Kip1^-expressing MEFs results in a dramatic up-regulation of EGFR expression, suggesting that p27^Kip1^-regulated EGFR expression is not only operative in bladder cancer cells, but also in non-bladder cancer and non-cancer cells. Fourthly, the levels of phosphorylation and nuclear translocation of the JNK and c-Jun transcriptional axis, whose interaction with the proximal promoter of the *Egfr* gene is essential for its gene transcription, are significantly higher in p27^Kip1−/−^ cells than in p27^Kip1+/+^ cells. Chemical inhibitors and dominant-negative mutants of JNK and c-Jun specifically repress the transcription of the *Egfr* gene in p27^Kip1^-deficient cells, and the recruitment of c-Jun to the *Egfr* proximal promoter in the absence of these inhibitors is much greater in p27^Kip1−/−^ cells than in p27^Kip1+/+^ cells. Finally, reintroduction of p27^Kip1^ into p27^Kip1^-deficient T24T cells markedly represses the activation of JNK and c-Jun and reduces EGFR expression. Taken together, these data firmly establish a signalling cascade linking p27^Kip1^ deficiency to EGFR overexpression through JNK/c-Jun transcriptional activation.

The results of the present study have important clinical implications on several fronts. The first relates to an improved understanding of the cellular functions of p27^Kip1^ in tumour suppression that are independent of its canonical role in CDK inhibition [33]. In normal resting cells, p27^Kip1^, expressed at high levels, hypo-phosphorylated and localized primarily in the nuclei, binds to CDK4–cyclin D and CDK2–cyclin E/A–CDK2 complexes, thereby functionally inactivating CDKs [34]. This is commensurate with the role of p27^Kip1^ in G_1_ arrest and tumour suppression [1,6,35]. Indeed, mice globally lacking p27^Kip1^ are significantly heavier than their wild-type counterparts because of multi-organ hyperplasia [35,36]. Although spontaneous tumours are uncommon in the p27^Kip1−/−^ mice [36], they are highly prone to tumorigenesis when exposed to chemical carcinogens [37]. These and other data from mouse models are therefore strongly supportive of the CDK-dependent activities of p27^Kip1^ in suppressing tumour formation *in vivo* [38]. Whether p27^Kip1^ exerts other tumour suppressive effects that can account for the phenotypes observed in the knockout mice has not been carefully addressed. Interestingly, there was a report showing that ablating the CDKs in p27^Kip1^-deficient cells does not completely abolish the proliferative ability of the host cells [39], raising the possibility that p27^Kip1^ can regulate a broader spectrum of downstream targets beyond the CDKs. On the basis of the recent characterization of gene-knockout mice, c-Jun integrates signals of several developmental pathways, including EGFR/ERK (extracellular-signal-regulated kinase) [40] and EGFR/RhoA/ROCK (Rho-associated kinase). To our current knowledge, c-Jun remains at the centre stage of a complex molecular network, c-Jun interacting with many signalling pathways, some of which are yet to be discovered. As we have demonstrated in the present study, one such CDK-independent p27^Kip1^-regulated downstream target is EGFR.

EGFR is a key member of the receptor tyrosine kinase family, and is activated in response to growth stimulators such as growth factors, cytokines and growth hormones [41]. It plays a diverse role in cell proliferation, angiogenesis, motility, invasion and metastasis, and overexpression of EGFR is therefore considered oncogenic for many cell types [42]. Different deubiquitinating enzymes may be critical in balancing c-Cbl and SMURF2 activity [43], thus tightly regulating EGFR protein stability, alteration of which during oncogenesis leads to EGFR overexpression. However, there is no significant difference between c-Cbl and SMURF2 in p27Kip1^+/+^ and p27Kip1^−/−^ MEFs (results not shown), suggesting that EGFR protein stability could be regulated independently of c-Cbl and SMURF2. Further to demonstrating the link between p27^Kip1^ and EGFR, we propose that the deficiency in p27^Kip1^ can lead to a synergistic effect on cell proliferation, one via the loss of CDK-dependent cell-cycle inhibition and another via EGFR overexpression. This is another example where a tumour suppressor can intersect with an oncogenic event to drive tumour formation although, in this case, the deficiency of one protein can lead to the dual effects. With respect to tumour progression, we wish to point out that EGFR overexpression only occurs in p27^Kip1^-deficient metastatic T24T cells, but not in the parental T24 cells which express p27^Kip1^ and are incapable of metastasizing. Although the role of EGFR overexpression in cancer progression is well established [44], the role of p27^Kip1^ is much less clear. In fact, increased cytoplasmic translocation of p27^Kip1^ has been associated with increased cell motility and invasion [45]. It has previously been reported that inhibiting Ser^10^ phosphorylation of p27 led to p27 accumulation in the nucleus and enhanced erlotinib-mediated cytotoxicity in breast cancer [46]. Therefore the net effect of p27^Kip1^ loss and EGFR overexpression on cancer cell metastasis needs to be further investigated [47].

Inhibition of JNK activation by pretreatment of T24T cells with the JNK inhibitor SP600125 blocked c-Jun phosphorylation at Ser^63^/Ser^73^ and anchorage-independent growth of T24T cells. This is consistent with the report by Cáceres et al. [48] that pretreatment with either SP600125 or the expression of a dominant-negative mutant JNK leads to down-regulation of EGFR phosphorylation and inhibits the invasive capacity of cancer cells. This notion is also supported by the results of Li et al. [49] which suggest that EGFR is involved in constitutive JNK activation in diffuse gliomas and that the ability to inhibit JNK activation might confer increased sensitivity to therapeutic modalities targeting this pathway.

A key point is how p27^Kip1^ might inhibit JNK activity. Our previous work indicated that p27^Kip1^ might target the Akt pathway which contributes to deactivation of the MKK7 (MAPK kinase 7)/JNK pathway [18]. It should be noted that overexpression of EGFR occurs in a wide range of human tumour tissues and, as with a reduced p27^Kip1^, is strongly associated with late-stage tumours and poor prognosis [5,50]. Thus far, few studies have examined the concordance between reduced p27^Kip1^ expression and increased EGFR expression, a topic that should be explored based on the information provided by present work [51]. Similarly, it will be interesting to revisit whether EGFR is overexpressed in p27^Kip1^-deficient mice and whether EGFR inhibition will render these mice resistant to tumorigenesis even after carcinogen treatment. Finally, the fact that p27^Kip1^-deficient cells overexpress EGFR raises the interesting possibility that tumours deficient in either or both tumour suppressors could benefit from a therapeutic approach combining chemotherapeutics with EGFR inhibitors [46]. The present study has demonstrated that p27^Kip1^ suppresses JNK/c-Jun activation and EGFR expression in mouse MEFs and human bladder cancer cells, and the results obtained are consistent with those from human cancer specimens. The results provide new insight into p27^Kip1^ suppressing cancer cell growth, migration and metastasis.
